# Bioinformatic Analysis and Tissue-Specific Expression of Clock Genes and *Opn5* in the Chinese Giant Salamander (*Andrias davidianus*) Under Different Light Conditions

**DOI:** 10.3390/ani16193118

**Published:** 2026-10-04

**Authors:** Biao Chen, Qin Zhou, Zhizhi Jing, Dong Yao, Jing Chen, Xianwen Zhou, Pei Wang

**Affiliations:** 1 College of Life Sciences, Jishou University, Jishou 416000, China; 2 Hunan Engineering Laboratory for Chinese Giant Salamander’s Resource Protection and Comprehensive Utilization, College of Life Sciences, Jishou University, Jishou 416000, China; 3 Affair Center of Animal Husbandry and Aquaculture in XiangXi Autonomous Prefecture, Jishou 416000, China

**Keywords:** Chinese giant salamander, light exposure, circadian rhythm, *Opn5*, central and peripheral tissues

## Abstract

Circadian rhythms are essential for physiological regulation and environmental adaptation of animals. Light, as a primary exogenous synchronizing signal, significantly influences the maintenance and remodeling of the circadian system. In vertebrates, circadian rhythms depend on the synergistic action of central and peripheral clocks; however, systematic studies on the molecular responses to light across different tissues in amphibians are still lacking. In this study, 64 juvenile Chinese giant salamanders (*Andrias davidianus*) were randomly assigned to four groups: a control group (approximately 12L:12D, 5–12 lx, C), a continuous intermediate-light group (24L:0D, 80 ± 5 lx, I), a continuous high-light group (24L:0D, 220 ± 10 lx, H), and a continuous dark group (0L:24D, 0 lx, B). The experiment spanned 13 days. We first identified candidate *Clock* and *Opn5* homologues from transcriptome-derived sequences using homology searches, phylogenetic analysis and conserved-domain analysis. We then used RT-qPCR to measure expression changes across sampling days of *Clock*, *Bmal1*, *Per2*, *Cry1* and *Opn5* in the brain, eye and skin. The results indicated that under control and continuous intermediate-light conditions, the expression changes in core clock genes in brain and eye tissues were generally consistent, while the skin exhibited a relatively independent response pattern. Under continuous high-light conditions, the brain and eye exhibited distinct temporal expression patterns, whereas constant darkness was associated with a later-stage transcriptional response in the skin. In summary, prolonged exposure to bright light and continuous darkness were associated with distinct tissue-specific and time-dependent transcriptional responses of *Opn5* and core clock genes in the brain, eye, and skin of juvenile Chinese giant salamanders. This study provides foundational data on the molecular responses of the clock genes and non-visual opsin in amphibians to abnormal lighting conditions.

## Simple Summary

Light is a key environmental cue that regulates daily biological rhythms in animals. However, how different body tissues respond to light conditions in amphibians remains poorly understood. In this study, we examined how clock-related and light-responsive genes respond to different light environments in the brain, eye, and skin of juvenile Chinese giant salamanders, an ecologically important and conservation-priority species. We found that each tissue showed a unique response pattern: certain clock genes in the brain and eye increased early under normal and intermediate light, but constant bright light suppressed these responses in the eye, whereas higher expression of some clock genes occurred later in the brain. The skin showed increased activity of clock genes late in the experiment under constant darkness. These findings indicate that different tissues of the Chinese giant salamander adjust their clock-related gene activity in distinct ways when the light environment changes. Our results contribute to our understanding of how amphibians perceive and respond to light at the molecular level.

## 1. Introduction

Diurnal cycles are among the most stable temporal cues in natural environments. To anticipate and adapt to these recurring changes, organisms from cyanobacteria to mammals have evolved endogenous circadian clocks [1,2]. In vertebrates, this timing system is driven mainly by a Transcriptional–Translational Feedback Loop (TTFL). CLOCK and BMAL1 form heterodimers that bind E-box elements and activate transcription of *Per1*, *Per2*, *Per3*, *Cry1* and *Cry2*. The accumulated PER-CRY complex then feeds back and represses this transcriptional activity [3,4,5]. This molecular oscillator operates in both the central nervous system and multiple peripheral tissues, where it helps regulate sleep–wake cycles, endocrine function, body temperature and immunity over time [6,7].

The core molecular architecture of the TTFL is highly conserved across vertebrates. Nevertheless, the mechanisms by which different species perceive light and synchronize their circadian clocks with the external environment have diverged substantially over the course of evolution [8]. In mammals, photic entrainment is mediated primarily by intrinsically photosensitive retinal ganglion cells (ipRGCs), which transmit light signals via the retinohypothalamic tract to the suprachiasmatic nucleus (SCN) [9]. By contrast, teleosts and amphibians can retain direct light responsiveness in extraocular tissues, resulting in a more distributed photosensory input [10]. Studies in cavefish have shown that opsins can mediate light detection by peripheral clocks [10]. Isolated zebrafish organs, cells and early embryos also respond directly to light [11,12]. In *Xenopus laevis*, the eye, pineal complex and skin express multiple type II opsins [13]. The deep brain of Xenopus tadpoles can detect short-wavelength light even without ocular or pineal input and transmit this signal to motor pathways [14]. This diversity of photoreceptive mechanisms is also evident in salamanders. In larval tiger salamanders (*Ambystoma tigrinum*), ON retinal ganglion cells exhibit intrinsic photosensitivity independent of conventional photoreceptor input [15], while cone photoreceptors can coexpress multiple spectrally distinct opsins [16]. Beyond retinal photoreception, light-dependent regulation of clock-gene expression has also been demonstrated in amphibians. In cultured *X. laevis* melanophores, light–dark cycles induced temporal changes in *Per1*, *Per2* and *Bmal1* expression, while brief blue-light exposure increased *Per1* and *Per2* transcript levels [17]. Artificial night lighting has also been shown to alter tail regeneration and prey consumption in the salamander Plethodon cinereus [18], further indicating that light-related physiological responses in salamanders may differ from those characterized in teleost models. Together, these findings indicate that photoreception and circadian synchronisation in aquatic vertebrates may involve inputs from multiple tissues rather than a single central pathway.

Among nonvisual photoreceptors, OPN5, also known as neuropsin, is an important candidate for linking short-wavelength light detection to local physiological responses. OPN5 is a short-wavelength-sensitive G protein-coupled receptor (GPCR) involved in local light detection in the mammalian retina and avian deep brain [19,20,21]. In fish, Opn5m and Opn5m2 exhibit UV sensitivity and Gi coupling, and are expressed in both the retina and brain [22]. In medaka (*Oryzias latipes*), pituitary melanotrophs can directly detect short-wavelength light through Opn5m, thereby regulating hormone release and pigmentation [23]. Prolonged darkness alters nonvisual light input and peripheral-clock synchronisation in cavefish, while extraocular tissues continue to express several opsins, including *Opn5* [24,25], suggesting that the absence of light alters, but does not necessarily eliminate, these molecular pathways. In amphibians, studies of photoreception have historically focused largely on the eye and on classical visual-opsin genes, including *RH1*, *SWS1*, *SWS2* and *LWS*, which mediate retinal sensitivity across different spectral ranges [26]. By comparison, nonvisual opsins have received considerably less attention. Although neuropsins such as *Opn5*, *Opn6* and *Opn8* have been identified in the Xenopus retina, their physiological functions remain incompletely understood, and comparable evidence from caudate amphibians and extraocular tissues remains limited [13,27]. This is particularly relevant in artificial breeding and intensive aquaculture, where inappropriate lighting and artificial light at night (ALAN) may represent persistent environmental disturbances [28]. Several studies have shown that ALAN can alter amphibian activity and energy allocation and can also affect development and physiological homeostasis in aquatic vertebrates [29,30,31].

The Chinese giant salamander (*Andrias davidianus*) is the largest extant amphibian and belongs to an ancient lineage of high conservation value [32]. Previous studies have reported circadian body-colour changes in juveniles [33] and nocturnal activity rhythms in reproductive individuals [34], indicating that its phenotype and behaviour are closely associated with daily light conditions. Despite increasing evidence for multi-tissue photoreception in other amphibians, whether comparable light-responsive molecular mechanisms operate across different tissues in *A. davidianus* remains unclear. Therefore, the present study compared expression profiles of *Clock*, *Bmal1*, *Per2*, *Cry1* and *Opn5* across experimental days in the brain, eye and skin of juvenile Chinese giant salamanders under constant intermediate light, sustained bright light and constant-darkness conditions, to characterize the transcriptional responses of different tissues to altered light environments.

## 2. Materials and Methods

### 2.1. Experimental Animals and Light Treatments

Juvenile Chinese giant salamanders used in this experiment were obtained from a single culture batch. After disinfection with 0.1% compound iodine solution, the animals were placed in culture basins (41.5 cm × 29.5 cm). All individuals were acclimated for 1 week under the same ambient-light conditions (12L:12D, approximately 5–12 lx during the light phase and 0 lx during the dark phase). Following a one-week adaptation period, the formal experiment commenced. Four treatment groups were established: a control group (C; 12L:12D, 5–12 lx during the light phase, 0 lx during the dark phase), a constant intermediate-light group (I; 24L:0D, 80 ± 5 lx), a constant bright-light group (H; 24L:0D, 220 ± 10 lx) and a constant-darkness group (B; 0L:24D dark, 0 lx). The 5–12 lx in the C group is the natural variation in indoor ambient light, rather than a programmed intensity range. For the I and H treatments, illumination was provided by LED strips installed 23 cm above the water surface of the culture basins. To further characterize the light source, the spectral power distribution of the LED strips was subsequently measured over the wavelength range of 380–780 nm using a spectroradiometer (HP320; Hangzhou LCE Intelligent Detection Instrument Co., Ltd., Hangzhou, China), and the measured spectrum is provided in Figure S1. Illuminance was measured near the water surface at five positions within each basin (the four corners and the centre) using a handheld lux meter (Model S8608; Xiamen Meidemei Intelligent Technology Co., Ltd., Xiamen, China), and the mean of the five measurements was used as the representative illuminance for each basin.

A total of 64 healthy juveniles, approximately 1 year old at the beginning of the experiment (4.65 ± 0.20 g), were randomly assigned to the four groups (*n* = 16 per group), with each treatment consisting of three independent experimental basins. Water temperature was maintained at 19.0 ± 0.5 °C throughout the experiment. Water was replaced daily, and bloodworms were fed to the salamanders at 08:00 every 2 days. Brain, eye and skin tissues were collected on days 0, 2, 4, 8, 12 and 13. On Day 0, one individual per group was sampled prior to treatment initiation, yielding four pretreatment samples; three were randomly selected for RT-qPCR analysis (*n* = 3) and one was retained as a backup. At subsequent sampling points, one individual was collected from each basin, yielding three biological replicates per treatment. All samples were collected before feeding. The treatment period lasted 13 days. After the Day 12 sampling, the light sources in the I and H groups were switched off, and Day 13 samples were collected approximately 24 h after light withdrawal. All samples were snap-frozen in liquid nitrogen and stored at −80 °C.

### 2.2. Candidate Gene Screening and Bioinformatics Analysis

Candidate transcripts were obtained from previously generated Chinese giant salamander transcriptome data that had been assembled de novo with Trinity. Open reading frames were predicted using ORFfinder web version (National Center for Biotechnology Information, Bethesda, MD, USA; accessed on 2 February 2026) and translated into candidate protein sequences. Candidate proteins were then analysed using BLASTp homology searches on the NCBI Web BLAST server (National Center for Biotechnology Information, Bethesda, MD, USA; accessed on 2 February 2026). Homology annotation was based primarily on UniProt/Swiss-Prot, and conserved domains were identified using an NCBI Conserved Domain Search [35]. Representative vertebrate CLOCK, NPAS2 and opsin-family protein sequences were downloaded from NCBI. Multiple sequence alignment was performed with MAFFT in the NGPhylogeny.fr workflow, and low-quality or highly gapped regions were trimmed with BMGE [36]. Maximum-likelihood trees were constructed in MEGA 12 using the JTT model, and node support was assessed with 1000 bootstrap replicates. The CLOCK–NPAS2 tree was rooted at the midpoint, and the opsin-family tree was rooted using rhodopsin (RHO) as the outgroup.

To further confirm candidate-gene identity, we compared amino acid sequences from the relevant protein families and screened for diagnostic conserved sites. Three-dimensional structures were predicted using AlphaFold Server, powered by AlphaFold 3 (Google DeepMind; accessed on 2 February 2026). and visualised in PyMOL version 3.1.4.1 (Schrödinger, LLC, New York, NY, USA). Physicochemical properties of the candidate proteins were predicted with the ExPASy ProtParam web server (SIB Swiss Institute of Bioinformatics, Lausanne, Switzerland; accessed on 2 February 2026) [37]. Signal peptides, transmembrane helices, subcellular localisation and nuclear localisation signals were predicted using SignalP 6.0, TMHMM 2.0, the WoLF PSORT web server and the cNLS Mapper web server, respectively; the cNLS Mapper cut-off score was set to 4.0. All web-based tools were accessed on 6 February 2026.

### 2.3. RNA Extraction, cDNA Synthesis and RT-qPCR Analysis

Tissue samples were homogenised in TRIzol lysis reagent, and total RNA was extracted with the TransZol Up Plus RNA Kit (TransGen Biotech Co., Ltd, Beijing, China) according to the manufacturer’s instructions. RNA purity and concentration were measured with a NanoDrop Lite Plus (Thermo Fisher Scientific, Waltham, MA, USA) spectrophotometer, and RNA integrity was assessed by 1% agarose (Guangzhou Saiguo Biotech Co., Ltd., Guangzhou, China) gel electrophoresis. Qualified RNA (1 µg) was reverse-transcribed into cDNA using the HP All-in-One qRT Master Mix Kit (younggenbio, Kunming, China), and the cDNA was stored at −20 °C. Gene-specific primers for *Clock*, *Cry1*, *Opn5*, *Bmal1*, *Per2* and the reference gene *Gapdh* were designed from transcriptome sequences and synthesised by Sangon Biotech (Shanghai, China). Primer sequences are listed in Table 1. RT-qPCR was performed on a QuantStudio 5 real-time PCR system (Thermo Fisher Scientific, Waltham, MA, USA) using PerfectStart Green qPCR SuperMix (TransGen Biotech Co., Ltd, Beijing, China). Amplification specificity was evaluated by melting-curve analysis.

### 2.4. Data Processing and Analysis

Relative expression of target genes was calculated using the 2^−ΔΔCt^ method, with the Day 0 samples used as the common pretreatment reference, and data are presented as the mean ± standard deviation (SD). Differences among treatments at each post-treatment time point, and temporal differences within each treatment including the common Day 0 pretreatment reference, were analysed by one-way analysis of variance (ANOVA) followed by Tukey’s multiple-comparisons test. Tukey-adjusted *p* < 0.05 was considered statistically significant. The variation in *Gapdh* Ct values was assessed separately within each tissue using the original Ct values from all RT-qPCR samples. To examine expression changes across sampling days in the eye, brain, and skin under different light conditions, 2^−ΔΔCt^ values from days 0, 2, 4, 8, 12, and 13 were summarized by treatment and tissue. Means and SDs were calculated, and the six time points were plotted at equal angular intervals around a polar coordinate circle. Error bars were defined as max (mean − SD, 0) for the lower bound and mean + SD for the upper bound. To analyse relative expression patterns across tissues and light conditions, genes were grouped within each tissue. For each gene, the maximum mean expression value across all treatments and time points was set to 1, and the normalised expression value was used to map bubble size. The 2^−ΔΔCt^ values, calculated relative to the common Day 0 pretreatment reference, represented the fold changes. Upregulation thresholds were set at 1.5-, 2- and 3-fold; downregulation thresholds were set at 2/3, 1/2 and 1/3. Changes falling between 2/3-fold and 1.5-fold were considered below the 1.5-fold threshold. Statistical analyses were performed in GraphPad Prism 10.1.2. Figures were generated in R for Windows 4.4.1, and Adobe Illustrator 2025 was used only for graphical assembly and layout adjustment; raw values were not altered.

## 3. Results

### 3.1. Molecular Identification of Core Clock Genes and a Candidate Photoreceptor Gene

Based on the transcriptome data, six candidate sequences were selected: DN2530, DN4755, DN3015, DN368, DN11020 and DN31336 (Table 2). DN2530 encodes a 943-aa protein. Its best BLASTp hit was CLOCK from *Tiliqua scincoides* (identity = 80.12%, coverage = 89%, E = 0). DN4755 also encodes a bHLH-PAS domain-containing protein; however, its best match was PASD1 from *Lissotriton helveticus* (identity = 76.09%, E = 0), and it was therefore excluded from further analysis as a *Clock* or *Npas2* homologue. DN368, DN11020 and DN3015 showed high sequence identity with BMAL1, PER2 and CRY1, respectively (identity > 85%, E = 0), and were annotated as candidate *Bmal1*, *Per2* and *Cry1* sequences. DN31336 encodes a 353-aa protein whose best match was OPN5 from *Ambystoma mexicanum* (identity = 85.31%, coverage = 100%, E = 0). The predicted protein contains the typical seven-transmembrane domain characteristic of opsins and exhibits conserved OPN5/neuropsin features; it was therefore annotated as a candidate *Opn5* sequence.

**Figure 1:**
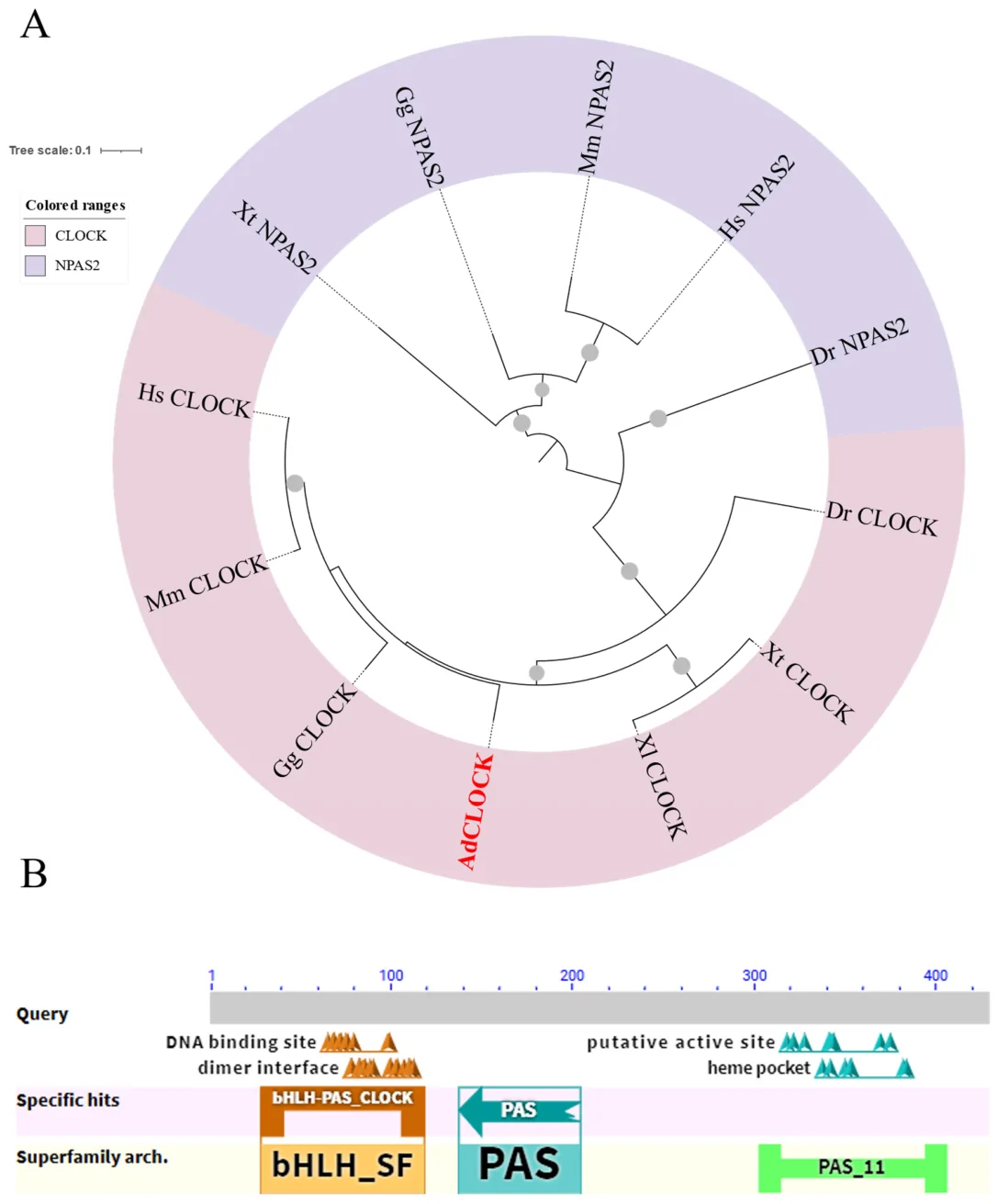
Phylogenetic placement and conserved domain composition of the Chinese giant salamander candidate *Clock* sequence DN2530 (Ad*Clock*). Notes: (**A**) Maximum-likelihood tree constructed from representative vertebrate CLOCK and NPAS2 protein sequences. AdCLOCK (DN2530) is highlighted in red. Bootstrap support values (1000 replicates) are indicated by circle sizes; filled circles indicate support values ≥ 80%, with larger circles representing higher support. (**B**) Conserved domain composition of the DN2530 protein. This protein contains a canonical bHLH–PAS_CLOCK domain, followed by PAS-A and PAS-B conserved regions.

CLOCK and its paralog NPAS2 both belong to the bHLH-PAS transcription factor family and show high sequence and domain similarity. We therefore verified the ortholog assignment of DN2530. Phylogenetic analysis showed that DN2530 belonged to the CLOCK clade, clearly separated from the NPAS2 clade (Figure 1A). Conserved Domain Database (CDD) annotation revealed that the predicted protein contained bHLH-PAS_CLOCK, PAS-A and PAS-B domains (Figure 1B). Multiple sequence alignment further identified six conserved sites that distinguish CLOCK from NPAS2; at all six sites, DN2530 matched the CLOCK consensus sequence (Table 3). Taken together, the phylogenetic position, domain composition and conserved residues support annotation of DN2530 as a *Clock* homologue in the Chinese giant salamander.

**Figure 2:**
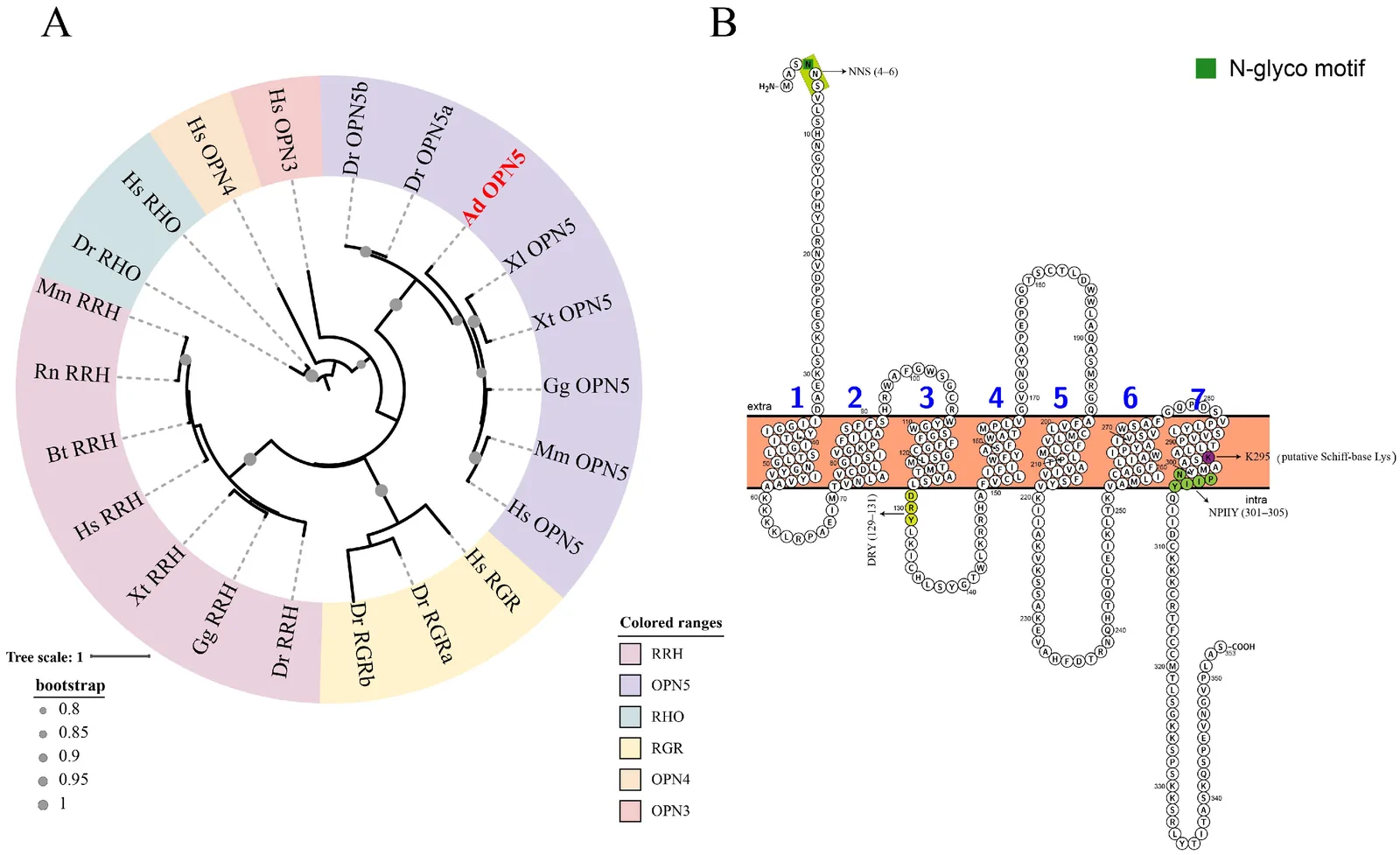
Phylogenetic placement and structural features of the Chinese giant salamander candidate *Opn5* sequence DN31336 (AdOPN5). Note: (**A**) Maximum-likelihood phylogenetic tree constructed from representative opsin-family protein sequences. Different background colors indicate different opsin subfamilies, and branch support values are indicated by circles of different sizes. DN31336(AdOPN5) is highlighted in red. (**B**) Predicted transmembrane topology of the DN31336 protein. Numbers 1–7 denote the seven predicted transmembrane helices. Yellow, purple and green highlights indicate the conserved DRY motif, the putative retinal-binding lysine residue K295 and the NPIIY motif, respectively. The protein possesses a canonical seven-transmembrane domain characteristic of rhodopsin-like GPCRs and contains conserved OPN5/neuropsin sequence motifs.

In the maximum-likelihood opsin-family tree rooted with RHO as the outgroup, DN31336 (AdOPN5) clustered within the OPN5 clade and was clearly separated from OPN3, OPN4, RRH and RGR (Figure 2A). InterPro annotation showed that DN31336 possesses the canonical seven-transmembrane domain characteristic of rhodopsin-like GPCRs and conserved OPN5/neuropsin motifs (Figure 2B). Its phylogenetic position and structural features support annotation of DN31336 as an *Opn5* homologue in the Chinese giant salamander.

### 3.2. Physicochemical Properties, Localisation Prediction, and Structural Modelling of Candidate Proteins

The physicochemical properties, transmembrane features, and predicted subcellular localisations of the five candidate proteins are summarised in Table 4. CLOCK, BMAL1, PER2 and CRY1 all lacked predictable signal peptides or transmembrane helices and exhibited negative GRAVY scores. By contrast, OPN5 contained seven predictable transmembrane helices and had a positive GRAVY value, displaying the hydrophobic transmembrane profile characteristic of typical opsins. WoLF PSORT predicted CLOCK, BMAL1, PER2 and CRY1 as predominantly nuclear proteins, whereas OPN5 was predominantly predicted to localise to the plasma membrane. The former four proteins each contained multiple predicted nuclear localisation signals (NLSs), consistent with their functional roles in circadian regulation in the nucleus. OPN5 also contained several predicted NLSs motifs; however, its seven-transmembrane topology and plasma membrane prediction are more consistent with the characteristics of a membrane-bound receptor. As these localisation features were inferred from in silico predictions, the subcellular localisation of OPN5 requires further experimental validation.

**Figure 3:**
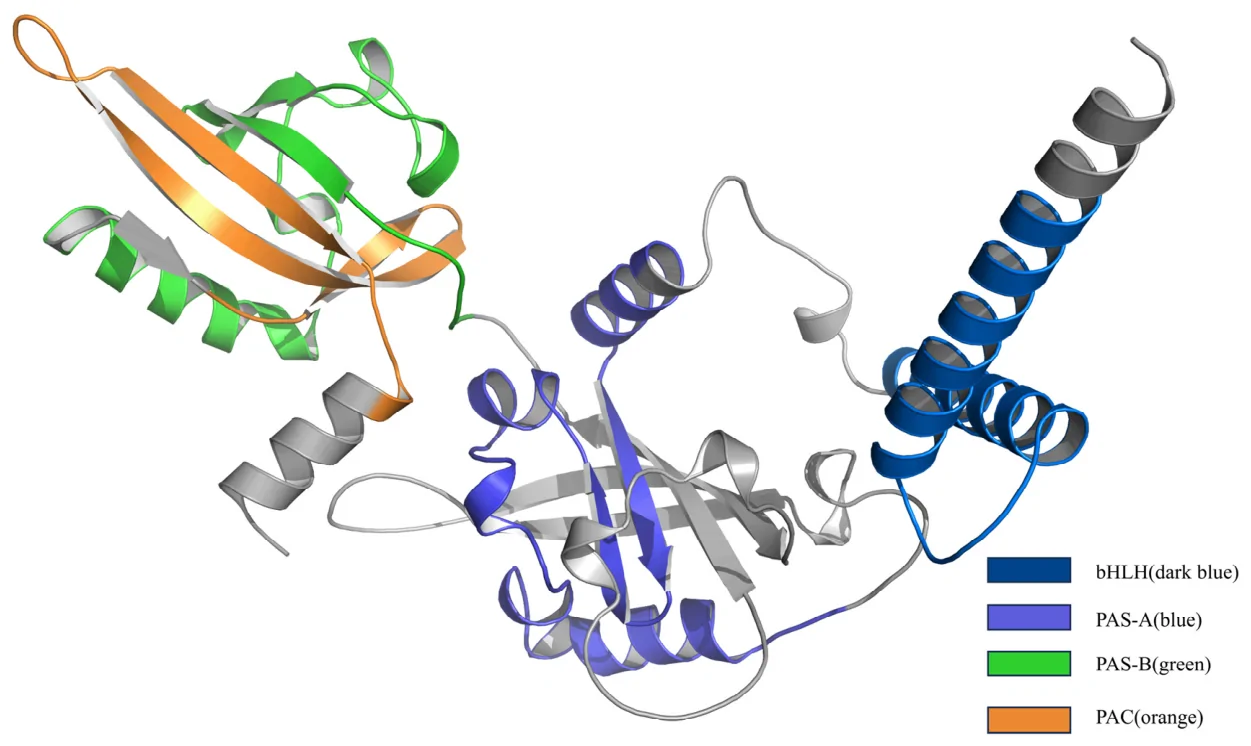
Predicted three-dimensional structure of the Chinese giant salamander CLOCK protein (DN2530). Note: The three-dimensional structure of the core functional region of the Chinese giant salamander CLOCK protein (residues 60–420), encompassing the bHLH, PAS-A, PAS-B and PAC domains was modelled using AlphaFold. Individual domains are shown in different colours.

To further evaluate the structural plausibility of the candidate CLOCK sequence, we used AlphaFold to model its core functional region (residues 60–420, encompassing the bHLH, PAS-A, PAS-B, and PAC domains). The predicted model exhibited a typical bHLH helix–loop–helix fold, dual PAS-A and PAS-B domains, and a short PAC region. The spatial arrangement of each domain was broadly consistent with the boundaries defined by sequence-based annotation, providing additional supporting evidence for the annotation of DN2530 as a CLOCK homologue (Figure 3).

### 3.3. Expression Changes Across Sampling Days of a Photoreceptor Gene and Core Clock Genes Under Different Light Treatments

**Figure 4:**
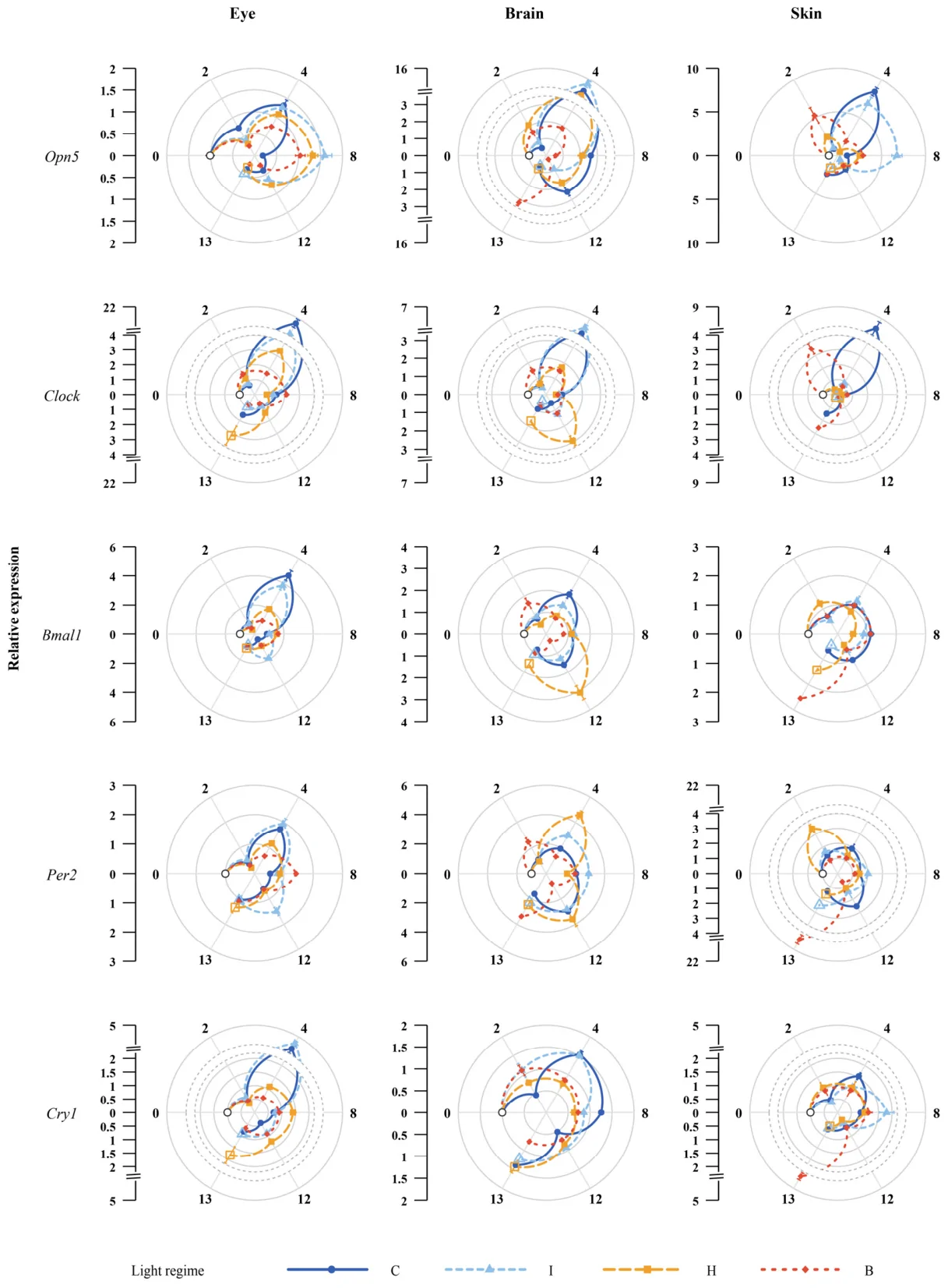
Expression changes across sampling days of *Opn5* and core clock genes in eye, brain and skin under different light conditions. Note: Each polar plot shows the temporal expression profile of one gene in one tissue under the four light treatments (C, I, H, and B). Different line styles and colours distinguish the four treatments. Curves and error bars indicate mean values and standard deviations (SDs), respectively. Radial axes begin at zero at the centre. Scale ranges differ among panels. Open symbols indicate the Day 13 observations for the I and H groups, which were collected approximately 24 h after cessation of constant illumination, distinguishing these points from those obtained during the main continuous-light treatment period.

Prior to analyzing target gene expression dynamics, Ct variation in the reference gene *Gapdh* was assessed across different tissues, light treatments, and sampling days based on the variation in original Ct values. *Gapdh* showed relatively limited expression variation across all conditions (CV < 5%, SD < 1.0 Ct), supporting its use as a reference gene for subsequent quantitative analyses, although this interpretation should be made cautiously because only a single reference gene was evaluated (Figure S2 and Table S1). Across fixed sampling points from days 0–13, *Opn5*, *Clock*, *Bmal1*, *Per2* and *Cry1* showed pronounced tissue-specific and treatment-dependent expression patterns in the eye, brain and skin (Figure 4).

In the eye, the expression patterns of *Clock*, *Bmal1* and *Cry1* were broadly similar between the control (C) and constant intermediate-light (I) groups: transcript levels of all three genes increased during days 2–4 and peaked on day 4. *Per2* also showed an early increase, albeit with a smaller amplitude. *Opn5* expression in the eye was generally low across all treatments and remained relatively stable over the sampling period. In the constant bright-light (H) and constant darkness (B) groups, the early increase in *Clock*, *Bmal1*, *Per2* and *Cry1* was attenuated compared with the C and I groups.

In the brain, *Opn5* showed the most pronounced temporal changes. In the C, I and H groups, *Opn5* expression increased to its highest levels during days 2–4, with the largest increase observed in the I group. In the B group, elevated *Opn5* expression occurred mainly on day 13. *Clock* and *Bmal1* showed relatively modest changes: both genes increased mainly during days 2–4 in the C and I groups, whereas higher expression was observed on days 8–12 in the H group. *Per2* fluctuated markedly at multiple time points. *Cry1* showed relatively small changes, with a modest early increase detected only in the C and I groups. The genes in C and I groups exhibited relatively similar temporal expression trends in their expression patterns.

The expression patterns in the skin differed from those in the eye and brain. In the C and I groups, *Opn5* expression increased mainly during days 2–8. In the H group, most genes remained relatively stable over the sampling period, only the expression level of *Per2* exhibited relatively pronounced fluctuations over time. In the B group, *Per2* increased on days 12–13, *Cry1* showed a similar late increase, and *Clock* and *Bmal1* displayed comparatively modest changes. In terms of expression patterns, *Opn5* and *Clock* exhibited similar profiles, with both reaching their highest expression levels on day 2. Likewise, *Bmal1*, *Per2*, and *Cry1* showed comparable expression patterns.

### 3.4. Relative Expression Patterns of Related Genes in Each Tissue Under Different Light Treatments

**Figure 5:**
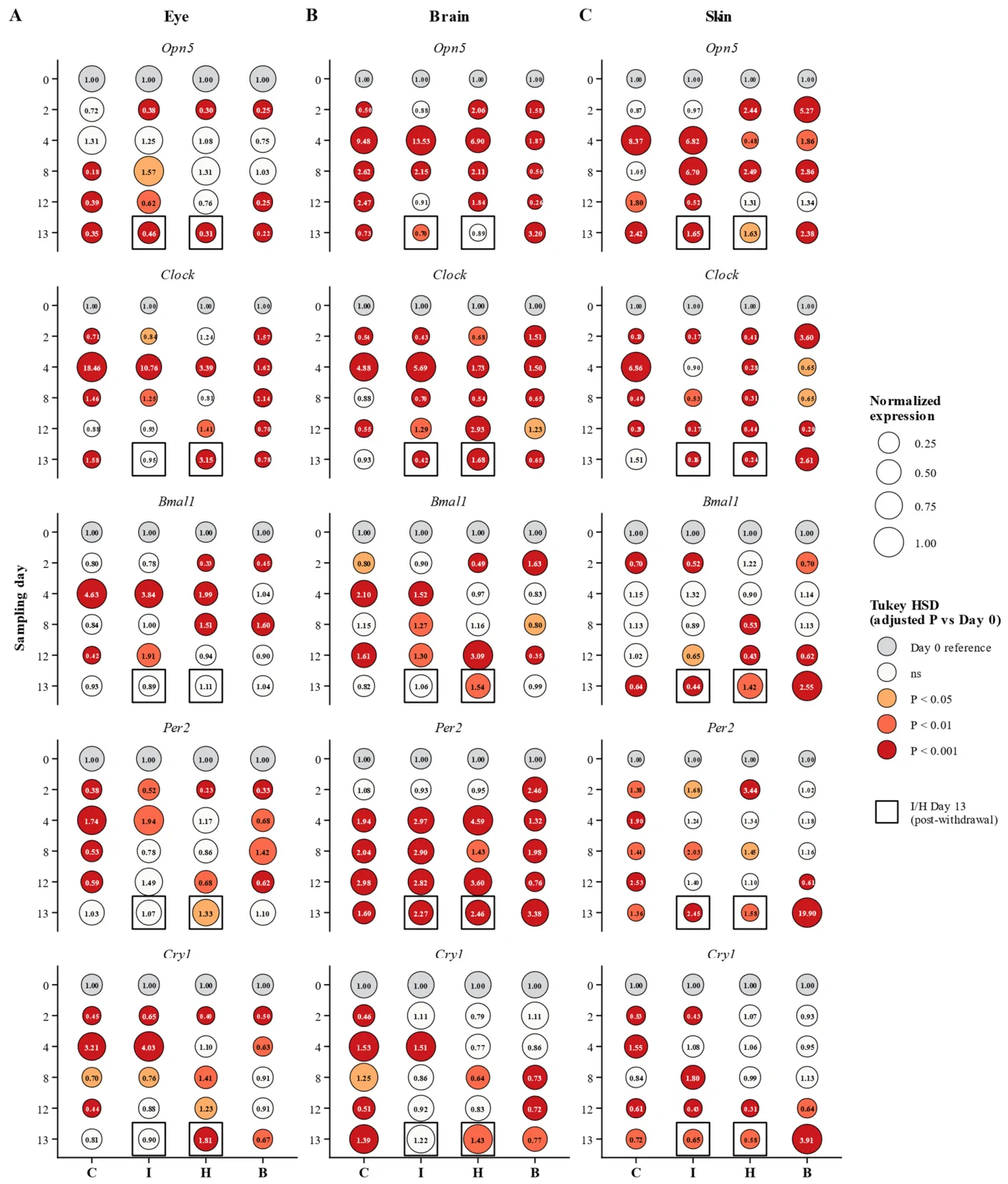
Relative expression patterns of *Opn5* and core clock genes in the eye, brain and skin under different light conditions. Note: (**A**) Eye; (**B**) brain; (**C**) skin. Within each tissue-gene panel, the maximum mean expression value of that gene across all treatments and time points was set to 1, and bubble size indicates normalised expression. Each bubble is labelled with the fold change relative to the common day 0 baseline (1.00). Fill colours indicate statistical significance relative to day 0 within the same tissue, gene and treatment. Black boxes indicate the Day 13 observations for the I and H groups, collected approximately 24 h after constant light was withdrawn, and distinguish these points from the primary continuous-light treatment period.

Using day 0 as a common baseline, fold changes in *Opn5* and the core clock genes showed tissues- and treatment-dependent differences in both timing and magnitude (Figure 5).

In the eye, upregulation of *Clock*, *Bmal1* and *Cry1* relative to day 0 was concentrated mainly on day 4 in the C and I groups. *Per2* was also upregulated early, but the magnitude was smaller and did not exceed the 2-fold threshold. *Opn5* did not reach the 1.5-fold upregulation threshold at most time points, and several values fell below the day 0 baseline. In the constant H and B groups, fewer core clock genes reached the upregulation 2-fold threshold, which only *Clock* on day 4, day 13 in H group, and on day 8 in B group were upregulation 2-fold threshold.

In the brain, *Opn5* showed the most prominent upregulation relative to day 0. In the C, I and H groups, *Opn5* reached its highest fold changes on day 4, whereas highest upregulation in the B group occurred mainly on day 13. *Clock* and *Bmal1* also showed time-dependent fold changes: upregulation in the C and I groups was concentrated mainly on day 4, whereas higher expression in the H group was observed mainly on day 12. *Per2* reached the upregulation threshold at several time points, and remained elevated over a relatively long interval. *Cry1* showed smaller fold changes and displayed early upregulation only in the C and I groups.

In the skin, *Opn5* reached the upregulation threshold on day 4 in the C and I groups. *Clock* was upregulated on day 4 in the C group and at several time points in the B group but showed limited changes under I and H. *Bmal1* showed limited overall fluctuation but was increased late under B group. In the B group, *Per2* reached its highest fold change on day 13, and *Cry1* was also upregulated during the late experimental period.

## 4. Discussion

### 4.1. Conserved Clock Transcription Factors and a Non-Visual Photoreceptor in the Chinese Giant Salamander

Vertebrate temporal regulation is typically maintained by a hierarchical system comprising central and peripheral clocks, rather than being governed exclusively by a single central oscillator [38]. Non-visual photoreception also contributes to photoentrainment by environmental light. In mice, *Opn5* deficiency impairs entrainment to specific photoperiods and delays readaptation following photoperiod shifts [39]. In the retina, violet light can induce the expression of light-responsive gene such as *Egr-1* through OPN5 [40], indicating that OPN5 can mediate local light responses. In the present study, homology searches, phylogenetic analysis, conserved-site comparison and domain analysis supported the sequence-based identification of the candidate *Clock* and *Opn5* homologs in the Chinese giant salamander. The candidate *Clock* sequence exhibited the canonical bHLH-PAS architecture, consistent with the conserved structural features of vertebrate CLOCK transcription factors [41]. The candidate *Opn5* sequence possessed the canonical opsin seven-transmembrane structure, consistent with the molecular basis of OPN5 as a non-visual photoreceptor [42]. OPN5 shows high sequence conservation and broad tissue functionality across vertebrates [43], supporting its use as a candidate molecule for analysing the link between environmental light input and tissue-specific transcriptional responses. The molecular identification results corroborate the presence of homologous sequences associated with *Clock* and *Opn5* in *A. davidianus* and provide a sequence basis for comparing expression changes across sampling days and tissues. Nevertheless, predictions based on sequences (inclusive of NLS analysis) are insufficient to ascertain the precise subcellular localization or functional attributes of OPN5, necessitating further experimental validation.

### 4.2. Tissue-Specific Transcriptional Responses in the Brain, Eye, and Skin

In the C and I groups, several core clock genes in eye were increased mainly during the early experimental period. In the brain, *Opn5* exhibited pronounced time-dependent variation under multiple light conditions. In the skin, prominent late-stage expression enhancement was observed under constant darkness. Together, these differences indicate that transcriptional responses of different tissues to altered light environments are not uniform in the Chinese giant salamander. The retina contains a local clock network formed by multiple cellular oscillators, enabling tissue-level circadian regulation in response to photic input [44]. In *X. laevis*, the eye, pineal complex, and skin express multiple type II opsins [13], providing direct molecular evidence for multi-tissue photoreception in amphibians. Furthermore, the deep brain of *X. laevis* tadpoles can also transmit light information directly to motor output [14], supporting the presence of non-visual light responsiveness in amphibian brain tissue. It is noteworthy that this experiment did not measure the gene expression levels in different brain regions; instead, it focused on the transcriptional profile of the entire brain tissue as a unified entity. The observed expression patterns should therefore be interpreted as integrated whole-brain responses rather than as evidence of phase relationships or synchronisation among regional brain clocks.

Nevertheless, we also observed that certain genes in both the eyes and brain displayed similar expression patterns under light–dark cycles (C group) and constant intermediate-light conditions (I group), although these comparisons should be interpreted cautiously because photoperiod and illuminance differed simultaneously between these two conditions. Furthermore, in the skin, the gene expression patterns of each treatment group demonstrated varying degrees of divergence from those of C group. Taken together, the distinct expression patterns of *Opn5* and the core clock genes in the brain, eye, and skin support a multi-tissue, tissue-specific transcriptional response to altered light environments in the Chinese giant salamander. Certainly, we are also cognizant of the fact that, this study only compared gene expression at fixed sampling points on different experimental days rather than continuously across a 24-h cycle, so the circadian rhythmicity, phase, amplitude, period, and inter-tissue phase relationships cannot be determined. In addition, the limited number of biological replicates at individual treatment–time combinations reduce statistical power, while the use of a single reference gene provides an additional source of uncertainty in individual comparisons. Accordingly, the results are interpreted primarily in terms of broader tissue- and treatment-related expression patterns across sampling days rather than isolated statistically significant differences at individual time points.

### 4.3. Divergent Transcriptional Responses of the Brain and Eye Under the Constant Bright Light

Under constant bright light, *Opn5* expression in the brain reached relatively high levels during the early experimental period, whereas the peak expression of *Clock* and *Bmal1* was observed during the middle to late period. This pattern suggests that constant bright light may be associated with time-dependent changes in the expression of clock-related genes in the brain, although it does not permit the inference that a specific regulatory pathway has been activated. In mouse skin, OPN5 mediates local light-dependent induction of clock genes and circadian photoentrainment [45]. In eye tissue, OPN5 also participates in light-dependent vascular development through dopamine-related pathway [46], indicating that its functions are markedly tissue-dependent. In teleost retina, a circadian link between *Clock* expression and cell proliferation-related genes has been reported [47], providing a cross-species precedent for local clock involvement in retinal light responses. Amphibian studies further support the possibility that the brain and eye process photic signals through distinct local mechanisms. In *X. laevis* tadpoles, a region of the caudal diencephalon retains direct sensitivity to short-wavelength light in the absence of ocular and pineal input, with OPN5-positive and CRY1-positive cells detected in the photosensitive region [14]. By contrast, the amphibian retina itself contains multiple light-responsive components. ON retinal ganglion cells in larval tiger salamanders (*Ambystoma tigrinum*) exhibit intrinsic photosensitivity [15], while isolated Xenopus retinal photoreceptor layers contain a local circadian oscillator whose phase can be reset directly by light [48]. These findings indicate that amphibian brain and retinal tissues possess distinct cellular substrates for responding to environmental light. Taken together with these findings, the early increase in *Opn5* expression and the later increase in *Clock* and *Bmal1* expression in the brain under constant bright light are best interpreted as time-dependent transcriptional adjustment. In the eye, by contrast, the attenuated early-expression peak and dispersed upregulation time points indicate that the brain and eye exhibit different transcriptional responses to constant bright light. Furthermore, no significant changes in gene expression were observed in the two continuous-light treatment groups (I and H) on Day 13, likely because the samples were collected 24 h after light cessation, by which time the acute transcriptional response to light removal may have subsided.

The divergent expression patterns observed in the brain and eye under constant bright light indicate that abnormal illumination can produce disparate molecular effects across tissues, although the available data are insufficient to determine whether such effects alter inter-tissue functional coordination. It should be noted that comparisons between the control and continuous-light groups reflect combined effects of altered photoperiod and illuminance, because the C group differed from the I and H groups in both light–dark cycle and illumination intensity. In contrast, comparisons between the two continuous-light groups may mainly reflect responses associated with different illumination intensities under the same 24L:0D photoperiod. Previous studies in other amphibians have also shown that artificial light at night can affect tail regeneration and feeding in salamanders and induce oxidative stress across developmental stages [18,49]. In artificial cultivation, *A. davidianus* is typically reared in dark caves. Although outdoor eco-mimicry cultivation methods also exist, they are relatively uncommon [50]. In this study, continuous intermediate-light conditions appeared to elicit a gene response pattern more similar to that of the control group, whereas the continuous darkness group exhibited a distinct expression pattern compared to the control. It is hypothesized that appropriate light factors can more effectively induce the normal expression of relevant genes. Simultaneously, it is crucial to acknowledge that the spectral characteristics under ambient lighting conditions were not assessed in this experiment. Consequently, the spectral discrepancies between LED treatment and ambient light control may offer insights into explaining the variations observed in transcriptional responses. These hypotheses necessitate further experimental validation. Currently, available Chinese giant salamanders breeding industry information does not establish standardized thresholds for illuminance, spectral composition, or photoperiod. Therefore, the current research findings merely offer molecular evidence regarding the responses to altered lighting conditions. A more comprehensive evaluation, incorporating relevant indicators such as behavior, physiology, and production, is essential to establish a robust foundation for determining specific lighting parameters in the *A. davidianus* aquaculture industry.

### 4.4. Late-Stage Transcriptional Adjustment in the Skin Under Constant Darkness

Under constant darkness, *Per2* expression in the skin increased during the late experimental period, *Cry1* showed a similar late increase, and *Clock*, *Bmal1* and *Opn5* exhibited comparatively modest changes. This pattern indicates that prolonged absence of light is associated with late-stage transcriptional adjustment in the skin. In *X. laevis*, environmental light can regulate skin pigmentation responses through type II opsins and melatonin receptors [51], indicating that amphibian skin is capable of receiving light-related signals. Cultured skin cells have been shown to exhibit circadian and damage-related responses to blue light [52], and the role of non-visual opsins in extraocular tissues has been supported by cumulative evidence [53]. The results of the present study are best interpreted as evidence of late-stage expression changes in skin clock-related genes under constant darkness, rather than as confirmation of enhanced autonomous clock function in the skin.

These results must be interpreted within the constraints of the present experimental design. Comparisons of daily expression at fixed time points cannot demonstrate endogenous circadian oscillations in the skin, nor can they establish phase coupling among the brain, eye, and skin. Accordingly, what constant darkness supports is altered expression of circadian-related genes and *Opn5* in the skin, rather than enhanced peripheral autonomous clock function. Moreover, feeding schedule may represent an additional non-photic synchronizing cue influencing physiological and transcriptional states. Future studies with controlled feeding conditions may further evaluate its contribution to temporal gene-expression patterns.

Future work should combine continuous high-frequency sampling across a complete 24 h cycle with protein quantification and tissue localisation analyses. This approach would allow assessment of period, amplitude and phase in each tissue, as well as the spatial relationship between OPN5 and core clock molecules. Additional experiments using multiple wavelengths, illuminance gradients and functional perturbation could help distinguish the roles of different light-sensing pathways in the eye, brain and skin and link molecular changes to behavioural and physiological outcomes.

## 5. Conclusions

In this study, we identified homologous sequences of *Clock* and *Opn5* in the Chinese giant salamander and compared the expression changes across sampling days of *Opn5* and core clock genes in the brain, eye, and skin of juveniles under different light conditions. In the control and constant intermediate-light groups, several core clock genes in the brain and eye increased mainly during the early experimental period. Constant bright light attenuated the early expression peak in the eye and shifted higher expression of certain clock genes towards the later period in the brain. Constant darkness was associated with late-stage upregulation of *Per2* and *Cry1* in the skin. These results support that juvenile Chinese giant salamanders exhibit tissue-specific and time-dependent transcriptional responses to altered light environments and provide an experimental basis for studying amphibian nonvisual photoreception and optimising light environments in artificial conservation.

## Figures and Tables

**Table 1:** Primer sequences for qPCR assays of clock-related genes.

Gene	Primer Sequence (5′-3′)	Tm (°C)	Product Length (bp)
*Clock*	F: 5′-CTACGCCGGTAGCATGTGGG-3′	62.97	175
	R: 5′-GGGTCAGCTGTGCGTGAGTG-3′	63.65	
*Cry1*	F: 5′-CGCCAGTCGCCTTTGGGAAG-3′	64.01	183
	R: 5′-CCTCGTGCGCGACAATAGGC-3′	64.13	
*Opn5*	F: 5′-GGCTGAAGAGACGTCATGCCT-3′	62.44	150
	R: 5′-TCACGACGCCAAAGGGACTC-3′	62.43	
*Bmal1*	F: 5′-CTGCAACTTGAGCTGCCTGGT-3′	63.46	111
	R: 5′-GCGTGCCGCGAAACATACTCT-3′	63.88	
*Per2*	F: 5′-AGGGATGCGGTACCCCTTGG-3′	63.83	115
	R: 5′-CTGCGGCATGGCACTGTTCA-3′	63.96	
*Gapdh*	F: 5′-CACTGAGGAACAGGTGGTCT-3′	58.95	163
	R: 5′-ATGGCTCATCAGGTCCACAA-3′	59.01	

**Table 2:** Homology-based annotation of candidate transcripts in the Chinese giant salamander.

Transcript	Protein Length (aa)	BEST HIT	Identity (%)	Coverage (%)	E-Value	Key Conserved Features	Annotation
DN2530	943	*Tiliqua scincoides* CLOCK	80.12	89	0.0	bHLH + PAS-A + PAS-B	CLOCK
DN4755	751	*Lissotriton helveticus* PASD1	76.09	92	0.0	bHLH + PAS-A + PAS-B	PASD1
DN3015	539	*Ambystoma mexicanum* CRY1	87.05	97	0.0	Cryptochrome/photolyase (PHR) core + FAD-binding domain	CRY1
DN368	633	*Pleurodeles waltl* BMAL1	96.84	100	0.0	bHLH + PAS-A + PAS-B	BMAL1
DN11020	595	*Cynops pyrrhogaster* PER2	86.99	98	0.0	Period_C + predicted IDRs	PER2
DN31336	353	*Ambystoma mexicanum* OPN5	85.31	100	0.0	Opsin family 7TM (GPCR-A); OPN5/neuropsin signature	OPN5

**Table 3:** Conserved amino acid differences between CLOCK and NPAS2 proteins.

DN2530 Position (aa)	DN2530 Amino Acid	CLOCK Consensus Amino Acid	NPAS2 Consensus Amino Acid	Domain (CDD Interval)
152	L	L	I	PAS domain (138–204)
155	M	M	T	PAS domain (138–204)
165	E	E	D	PAS domain (138–204)
172	E	E	G	PAS domain (138–204)
352	K	K	R	PAS-like superfamily region (303–406)
394	R	R	K	PAS-like superfamily region (303–406)

**Table 4:** Physicochemical properties, transmembrane features and predicted subcellular localisation of candidate proteins.

Protein	Length (aa)	Mw (Da)	pI	Instability Index	GRAVY	SignalP	TMHMM	NLS	WoLF PSORT
CLOCK	943	105,886.2	6.94	63.03	−0.544	NO	0	1	nucl = 22.5
OPN5	353	39,320.58	9.48	35.83	0.413	NO	7	6	plas = 32
CRY1	539	61,733.99	9.22	36.9	−0.484	NO	0	4	nucl = 13.5
BMAL1	633	69,320.34	6.05	47.05	−0.445	NO	0	8	nucl = 31
PER2	595	63,937.13	5.56	69.94	−0.762	NO	0	3	nucl = 25

Note: Length, amino acid length; Mw, theoretical molecular weight; pI, theoretical isoelectric point; GRAVY, grand average of hydropathicity; SignalP, prediction of classical signal peptides; TMHMM, number of predicted transmembrane helices; NLS, number of predicted nuclear localisation signals; WoLF PSORT, predicted primary subcellular localisation. WoLF PSORT results are shown as the compartment with the highest score, where nucl indicates = nucleus and plas = plasma membrane.

## Data Availability

The data presented in this study are available within this article.

## Supplementary Materials

The following supporting information can be downloaded at https://www.mdpi.com/article/10.3390/ani16193118/s1: Figure S1: Normalized spectral irradiance distribution of the LED light source used in the light treatment experiments; Figure S2: Assessment of Gapdh Ct value variation under different experimental conditions based on raw Ct values; Table S1: Descriptive assessment of Gapdh Ct value variation across tissues under the present experimental conditions.

## References

[1] Roenneberg T., Merrow M. (2016). The circadian clock and human health. Curr. Biol..

[2] Pittendrigh C.S. (1993). Temporal organization: Reflections of a Darwinian clock-watcher. Annu. Rev. Physiol..

[3] Takahashi J.S. (2017). Transcriptional architecture of the mammalian circadian clock. Nat. Rev. Genet..

[4] Partch C.L., Green C.B., Takahashi J.S. (2014). Molecular architecture of the mammalian circadian clock. Trends Cell Biol..

[5] Reppert S.M., Weaver D.R. (2002). Coordination of circadian timing in mammals. Nature.

[6] Mohawk J.A., Green C.B., Takahashi J.S. (2012). Central and peripheral circadian clocks in mammals. Annu. Rev. Neurosci..

[7] Dibner C., Schibler U., Albrecht U. (2010). The mammalian circadian timing system: Organization and coordination of central and peripheral clocks. Annu. Rev. Physiol..

[8] Menaker M., Moreira L.F., Tosini G. (1997). Evolution of circadian organization in vertebrates. Braz. J. Med. Biol. Res..

[9] Do M.T., Yau K.W. (2010). Intrinsically photosensitive retinal ganglion cells. Physiol. Rev..

[10] Cavallari N., Frigato E., Vallone D., Fröhlich N., Lopez-Olmeda J.F., Foa A., Berti R., Sánchez-Vázquez F.J., Bertolucci C., Foulkes N.S. (2011). A blind circadian clock in cavefish reveals that opsins mediate peripheral clock photoreception. PLoS Biol..

[11] Whitmore D., Foulkes N.S., Sassone-Corsi P. (2000). Light acts directly on organs and cells in culture to set the vertebrate circadian clock. Nature.

[12] Tamai T.K., Vardhanabhuti V., Foulkes N.S., Whitmore D. (2004). Early embryonic light detection improves survival. Curr. Biol..

[13] Bertolesi G.E., Debnath N., Malik H.R., Man L.L.H., McFarlane S. (2022). Type II opsins in the eye, the pineal complex and the skin of *Xenopus laevis*: Using changes in skin pigmentation as a readout of visual and circadian activity. Front. Neuroanat..

[14] Currie S.P., Doherty G.H., Sillar K.T. (2016). Deep-brain photoreception links luminance detection to motor output in Xenopus frog tadpoles. Proc. Natl. Acad. Sci. USA.

[15] Rajaraman K. (2012). ON ganglion cells are intrinsically photosensitive in the tiger salamander retina. J. Comp. Neurol..

[16] Isayama T., Chen Y., Kono M., Fabre E., Slavsky M., DeGrip W.J., Ma J.-X., Crouch R.K., Makino C.L. (2014). Coexpression of three opsins in cone photoreceptors of the salamander *Ambystoma tigrinum*. J. Comp. Neurol..

[17] Moraes M.N.C.M., Poletini M.O., Ramos B.C.R., de Lima L.H.R.G., Castrucci A.M.L. (2014). Effect of light on expression of clock genes in *Xenopus laevis* melanophores. Photochem. Photobiol..

[18] Wise S.E., Rohacek A., Scanlon A.E., Cabrera T., Buchanan B.W. (2022). The effects of artificial night lighting on tail regeneration and prey consumption in a nocturnal salamander (*Plethodon cinereus*) and on the behavior of fruit fly prey (*Drosophila virilis*). Animals.

[19] Kojima D., Mori S., Torii M., Wada A., Morishita R., Fukada Y. (2011). UV-sensitive photoreceptor protein OPN5 in humans and mice. PLoS ONE.

[20] Nakane Y., Ikegami K., Ono H., Yamamoto N., Yoshida S., Hirunagi K., Ebihara S., Kubo Y., Yoshimura T. (2010). A mammalian neural tissue opsin (Opsin 5) is a deep-brain photoreceptor in birds. Proc. Natl. Acad. Sci. USA.

[21] Collin S.P., Davies W.L., Hart N.S., Hunt D.M. (2009). The evolution of early vertebrate photoreceptors. Philos. Trans. R. Soc. B Biol. Sci..

[22] Sato K., Yamashita T., Haruki Y., Ohuchi H., Kinoshita M., Shichida Y. (2016). Two UV-sensitive photoreceptor proteins, Opn5m and Opn5m2, in ray-finned fish with distinct molecular properties and broad distribution in the retina and brain. PLoS ONE.

[23] Fukuda A., Sato K., Fujimori C., Yamashita T., Takeuchi A., Ohuchi H., Umatani C., Kanda S. (2025). Direct photoreception by pituitary endocrine cells regulates hormone release and pigmentation. Science.

[24] Simon N., Fujita S., Porter M., Yoshizawa M. (2019). Expression of extraocular opsin genes and light-dependent basal activity of blind cavefish. PeerJ.

[25] Ceinos R.M., Frigato E., Pagano C., Fröhlich N., Negrini P., Cavallari N., Vallone D., Fuselli S., Bertolucci C., Foulkes N.S. (2018). Mutations in blind cavefish target the light-regulated circadian clock gene, period 2. Sci. Rep..

[26] Mohun S.M., Davies W.I.L. (2019). The evolution of amphibian photoreception. Front. Ecol. Evol..

[27] Man L.L.H., Storey S.S., Bertolesi G.E., McFarlane S. (2023). Cell-type expression and activation by light of neuropsins in the developing and mature Xenopus retina. Front. Cell. Neurosci..

[28] Gaston K.J., Bennie J., Davies T.W., Hopkins J. (2013). The ecological impacts of nighttime light pollution: A mechanistic appraisal. Biol. Rev. Camb. Philos. Soc..

[29] Perry G., Buchanan B.W., Fisher R.N., Salmon M., Wise S.E., Mitchell J.C., Jung Brown R.E., Bartholomew B. (2008). Effects of artificial night lighting on amphibians and reptiles in urban environments. Urban Herpetology.

[30] Touzot M., Teulier L., Lengagne T., Secondi J., Théry M., Libourel P.A. (2019). Artificial light at night disturbs the activity and energy allocation of the common toad during the breeding period. Conserv. Physiol..

[31] Falcón J., Torriglia A., Attia D., Viénot F., Gronfier C., Behar-Cohen F., Martinsons C., Hicks D. (2020). Exposure to artificial light at night and the consequences for flora, fauna, and ecosystems. Front. Neurosci..

[32] Browne R.K., Li H., Wang Z., Okada S., Hime P., McMillan A., Wu M., Diaz R., McGinnity D., Briggler J.T. (2014). The giant salamanders (Cryptobranchidae): Part B. Biogeography, ecology and reproduction. Amphib. Reptile Conserv..

[33] Zhang Y., Wang Z., Luo Q., Li H., Wang P., Wang J., Li D., Wang W., Yuan K., Zhou Y. (2025). Circadian rhythm of body color change in the juvenile Chinese giant salamander (*Andrias davidianus*) under different photoperiods. Animals.

[34] Luo Q., Song Y., Ji H. (2017). Nocturnal activity rhythm of Chinese giant salamander (*Andrias davidianus*) during the reproductive period. AMSE J. Model. C.

[35] Wang J., Chitsaz F., Derbyshire M.K., Gonzales N.R., Gwadz M., Lu S., Marchler G.H., Song J.S., Thanki N., Yamashita R.A. (2023). The conserved domain database in 2023. Nucleic Acids Res..

[36] Lemoine F., Correia D., Lefort V., Doppelt-Azeroual O., Mareuil F., Cohen-Boulakia S., Gascuel O. (2019). NGPhylogeny.fr: New generation phylogenetic services for non-specialists. Nucleic Acids Res..

[37] Gasteiger E., Hoogland C., Gattiker A., Duvaud S., Wilkins M.R., Appel R.D., Bairoch A., Walker J.M. (2005). Protein identification and analysis tools on the ExPASy server. The Proteomics Protocols Handbook.

[38] Patke A., Young M.W., Axelrod S. (2020). Molecular mechanisms and physiological importance of circadian rhythms. Nat. Rev. Mol. Cell Biol..

[39] Ota W., Nakane Y., Hattar S., Yoshimura T. (2018). Impaired Circadian Photoentrainment in *Opn5*-Null Mice. iScience.

[40] Jeong H., Lee D., Jiang X., Negishi K., Tsubota K., Kurihara T. (2023). Opsin 5 mediates violet light-induced early growth response-1 expression in the mouse retina. Sci. Rep..

[41] Wu D., Rastinejad F. (2017). Structural characterization of mammalian bHLH-PAS transcription factors. Curr. Opin. Struct. Biol..

[42] Yamashita T. (2020). Unexpected molecular diversity of vertebrate nonvisual opsin *Opn5*. Biophys. Rev..

[43] Calligaro H., Dkhissi-Benyahya O., Panda S. (2022). Ocular and extraocular roles of neuropsin in vertebrates. Trends Neurosci..

[44] Ko G.Y.P. (2020). Circadian regulation in the retina: From molecules to network. Eur. J. Neurosci..

[45] Buhr E.D., Vemaraju S., Diaz N., Lang R.A., Van Gelder R.N. (2019). Neuropsin (OPN5) mediates local light-dependent induction of circadian clock genes and circadian photoentrainment in exposed murine skin. Curr. Biol..

[46] Nguyen M.-T.T., Vemaraju S., Nayak G., Odaka Y., Buhr E.D., Alonzo N., Tran U., Batie M., Upton B.A., Darvas M. (2019). An opsin 5-dopamine pathway mediates light-dependent vascular development in the eye. Nat. Cell Biol..

[47] Song H., Wang D., Perez F.D.J., Xie R., Liu Z., Chen C.-C., Yu M., Yuan L., Fernald R.D., Zhao S. (2017). Rhythmic expressed clock regulates the transcription of proliferating cellular nuclear antigen in teleost retina. Exp. Eye Res..

[48] Cahill G.M., Besharse J.C. (1993). Circadian clock functions localized in Xenopus retinal photoreceptors. Neuron.

[49] Petrović T.G., Gavrić-Čampar J.P., Burraco P., Gavrilović B.R., Despotović S.G., Radovanović T.B., Radmilović A., Mirč M., Kolarov N.T., Prokić M.D. (2025). Developing under artificial light is not so bright: Oxidative stress as a physiological response to light pollution across amphibian life stages. Comp. Biochem. Physiol. A Mol. Integr. Physiol..

[50] Jiang W., Tian H., Zhang L., Seifert A.W., Currie J.D. (2023). Husbandry, captive breeding, and field survey of Chinese giant salamander (*Andrias davidianus*). Salamanders: Methods and Protocols.

[51] Bertolesi G.E., Atkinson-Leadbeater K., Mackey E.M., Song Y.N., Heyne B., McFarlane S. (2020). The regulation of skin pigmentation in response to environmental light by pineal type II opsins and skin melanophore melatonin receptors. J. Photochem. Photobiol. B.

[52] Dong K., Goyarts E.C., Pelle E., Trivero J., Pernodet N. (2019). Blue light disrupts the circadian rhythm and create damage in skin cells. Int. J. Cosmet. Sci..

[53] Andrabi M., Upton B.A., Lang R.A., Vemaraju S. (2023). An expanding role for nonvisual opsins in extraocular light sensing physiology. Annu. Rev. Vis. Sci..

