# Supplementary material for: Bioinformatic Analysis and Tissue-Specific Expression of Clock Genes and Opn5 in the Chinese Giant Salamander (Andrias davidianus) Under Different Light Conditions
Source: Animals (Basel). 2026 Oct 4;16(19):3118. doi: 10.3390/ani16193118 (PMC13663383; doi:10.3390/ani16193118)
Supplement: Supplementary file 1 [file animals-16-03118-s001.zip › animals-4456277-supplementary.pdf]

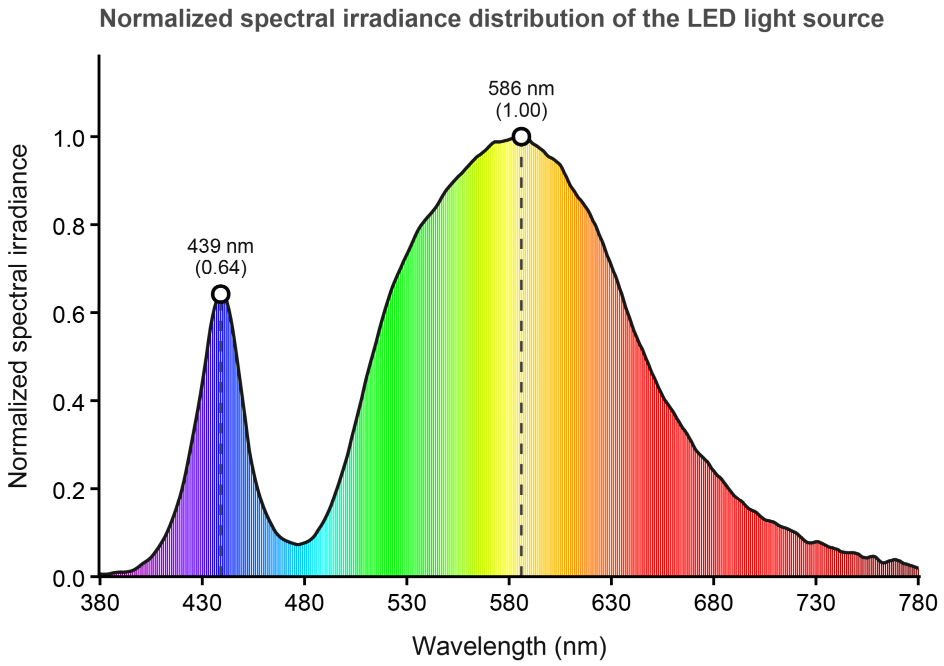

Figure S1. Normalized spectral irradiance distribution of the LED light source used in the light treatment experiments.

The spectral distribution of the LED light source was measured across wavelengths from 380 to 780 nm and normalized to the maximum spectral irradiance. The spectrum showed two major emission peaks at approximately 439 nm and 586 nm, with relative normalized irradiance values of 0.64 and 1.00, respectively. The measured spectral profile was used to characterize the light environment applied in the experimental treatments.

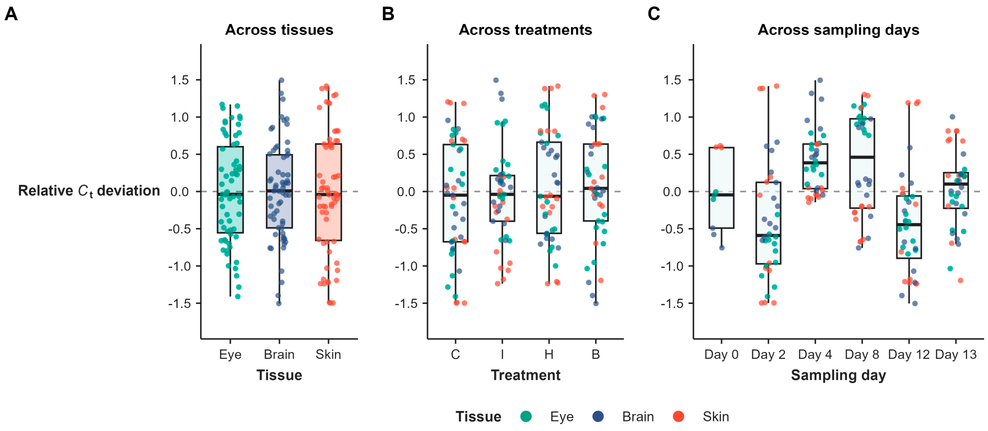

Figure S2. Assessment of *Gapdh* Ct value variation under different experimental conditions based on raw Ct values.

Raw Ct values of *Gapdh* were obtained before normalization. Because absolute Ct values may differ among tissues due to tissue-specific characteristics and differences in cDNA input, relative Ct deviation within each tissue was calculated as: $\text{Relative } C_t \text{ deviation}_i = C_{t,i} - \bar{C}_{t\,\text{same tissue}}$ where $C_{t,i}$ represents the raw Ct value of an individual sample, and $\bar{C}_{t\,\text{same tissue}}$ represents the mean Ct value of *Gapdh* within the corresponding tissue. Boxplots indicate the median and interquartile range, and individual points represent individual samples. This analysis was used to evaluate the within-tissue variation of *Gapdh* Ct values across different treatments and sampling days under the present experimental conditions.

Table S1. Descriptive assessment of *Gapdh* Ct value variation across tissues under the present experimental conditions

Tissue	Total measurements (n)	Mean Ct ± SD	CV (%)	Variation assessment
Eye	63	16.83 ± 0.69	4.07	Limited Ct variation (SD < 1.0 Ct)
Brain	63	14.37 ± 0.66	4.58	Limited Ct variation (SD < 1.0 Ct)
Skin	63	20.02 ± 0.83	4.12	Limited Ct variation (SD < 1.0 Ct)

Note: Ct variation was evaluated based on the SD and CV of *Gapdh* Ct values within each tissue across all analyzed samples. CV was calculated as SD/mean Ct × 100%. SD values below 1 Ct were considered indicative of relatively limited Ct variation. n represents the total number of Ct measurements obtained from all samples within each tissue.
